# Supplementary figures and images for: Effects of Combining a Ketogenic Diet with Resistance Training on Body Composition, Strength, and Mechanical Power in Trained Individuals: A Narrative Review
Source: Nutrients. 2021 Sep 1;13(9):3083. doi: 10.3390/nu13093083 (PMC8469041; doi:10.3390/nu13093083)

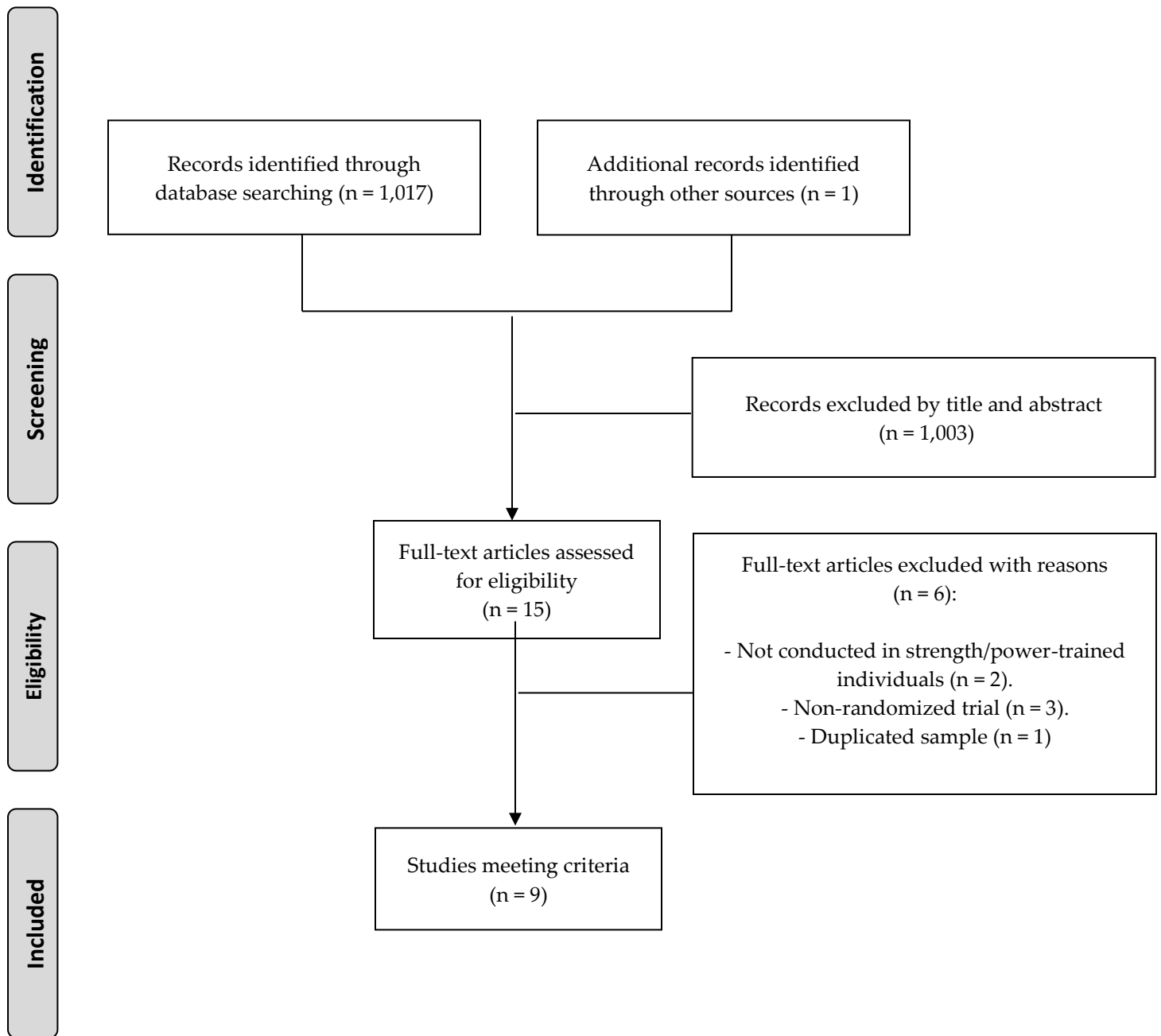

Supplement: Supplementary file 1 [file nutrients-13-03083-s001.zip › nutrients-1298439-supplementary.pdf]
